# Experimental study and simulation of phosphorus purification effects of bioretention systems on urban surface runoff

**DOI:** 10.1371/journal.pone.0196339

**Published:** 2018-05-09

**Authors:** Jiake Li, Zheng Liang, Yajiao Li, Peng Li, Chunbo Jiang

**Affiliations:** 1 State Key Laboratory of Eco-hydraulics in Northwest Arid Region of China, Xi’an University of Technology, Xi’an, China; 2 School of Architecture and Civil Engineering, Xi’an University of Science and Technology, Xi’an, China; Centre National de la Recherche Scientifique, FRANCE

## Abstract

Excessive phosphorus (P) contributes to eutrophication by degrading water quality and limiting human use of water resources. Identifying economic and convenient methods to control soluble reactive phosphorus (SRP) pollution in urban runoff is the key point of rainwater management strategies. Through three series of different tests involving influencing factors, continuous operation and intermittent operation, this study explored the purification effects of bioretention tanks under different experimental conditions, it included nine intermittent tests, single field continuous test with three groups of different fillers (Fly ash mixed with sand, Blast furnace slag, and Soil), and eight intermittent tests with single filler (Blast furnace slag mixed with sand). Among the three filler combinations studied, the filler with fly ash mixed with sand achieved the best pollution reduction efficiency. The setting of the submerged zone exerted minimal influence on the P removal of the three filler combinations. An extension of the dry period slightly promoted the P purification effect. The combination of fly ash mixed with sand demonstrated a positive purification effect on SRP during short- or long-term simulated rainfall duration. Blast furnace slag also presented a positive purification effect in the short term, although its continuous purification effect on SRP was poor in the long term. The purification abilities of soil in the short and long terms were weak. Under intermittent operations across different seasons, SRP removal was unstable, and effluent concentration processes were different. The purification effect of the bioretention system on SRP was predicted through partial least squares regression (PLS) modeling analysis. The event mean concentration removal of SRP was positively related to the adsorption capacity of filler and rainfall interval time and negatively related to submerged zones, influent concentration and volume.

## Introduction

The increase in impervious surfaces accompanying urban development has caused a rise in the volume of stormwater runoff and in the amount of phosphorus (P) pollution in urban runoff that reaches surface water [1,2]. Excessive P and nitrogen (N) in a water body can cause eutrophication by stimulating algal growth, degrading water quality and limiting human use of water resources [3–5]. Phosphorus is often the limiting element in water ecosystems. Phosphorus in urban runoff is distributed between P affiliated with particulate matter (particulate phosphorus, PP) and dissolved forms (dissolved phosphorus, DP). Dissolved phosphorus includes soluble reactive phosphorus (SRP, commonly assumed to be inorganic phosphate) and dissolved organic phosphorus (DOP). Soluble reactive phosphorus plays a more limited role in P transport in comparison with PP and DOP [6]. Identifying economic and convenient methods to control SRP pollution in urban runoff is the key point of rainwater management strategies.

Rainwater management strategies have evolved continuously through years of exploration and practice. Bioretention facilities are effective urban stormwater control measures (SCMs) to successfully manage flow volume and mitigate a multitude of pollutants [7–11]. During rainfall events, bioretention systems treat stormwater through a range of physical (e.g. sedimentation and filtration), chemical (e.g. adsorption, chemical precipitation and ion exchange) and biological processes (e.g. plant and microbial uptake) [12, 13].

P uptake via adsorption is controlled by the adsorption capacity of the media and the previous exposure history of the media to the adsorbate. With the accumulation and eventual saturation of P in the media, the media should gradually demonstrate P adsorption breakthrough and exhaustion [14]. Therefore, P treatment through bioretention is highly variable. In several cases, P concentration removal has been observed [8,15–21]. However, several studies have shown that when effluent concentrations are higher than influent concentrations [7,10,22–25], it is mainly due to the leaching of P from compost. It is noteworthy that excess amounts of P will cause eutrophication and groundwater contamination [26]. Therefore, leachates from compost in the soil or filler must be considered when designing bioretention systems [27].

Although many areas of P treatment through bioretention have gradually matured, this technology involves significant localisation conditions. A variety of internal and external conditions affect the results of bioretention operations [17,28]. Therefore, bioretention facilities are designed and applied based on local characteristics and conditions of rainfall, storm runoff, soil type, plant species and so forth. Numerous countries and regions have yet to create a bioretention design manual because of the lack of research data on the practical operation effect of bioretention. This situation affects the design and operation of bioretention facilities. Through pilot tests, the current research explores the internal and external influencing factors of P removal efficiency of bioretention and P removal performance under continuous and intermittent operation. It included nine intermittent tests, single field continuous test with three groups of different fillers (Fly ash mixed with sand, Blast furnace slag, and Soil), and eight intermittent tests with single filler (Blast furnace slag mixed with sand). The local characteristics and conditions of the Xi’an region are considered. A mathematical model of SRP removal performance and influencing factors is established.

## Materials and methods

### Test system devices

**Fig 1:**
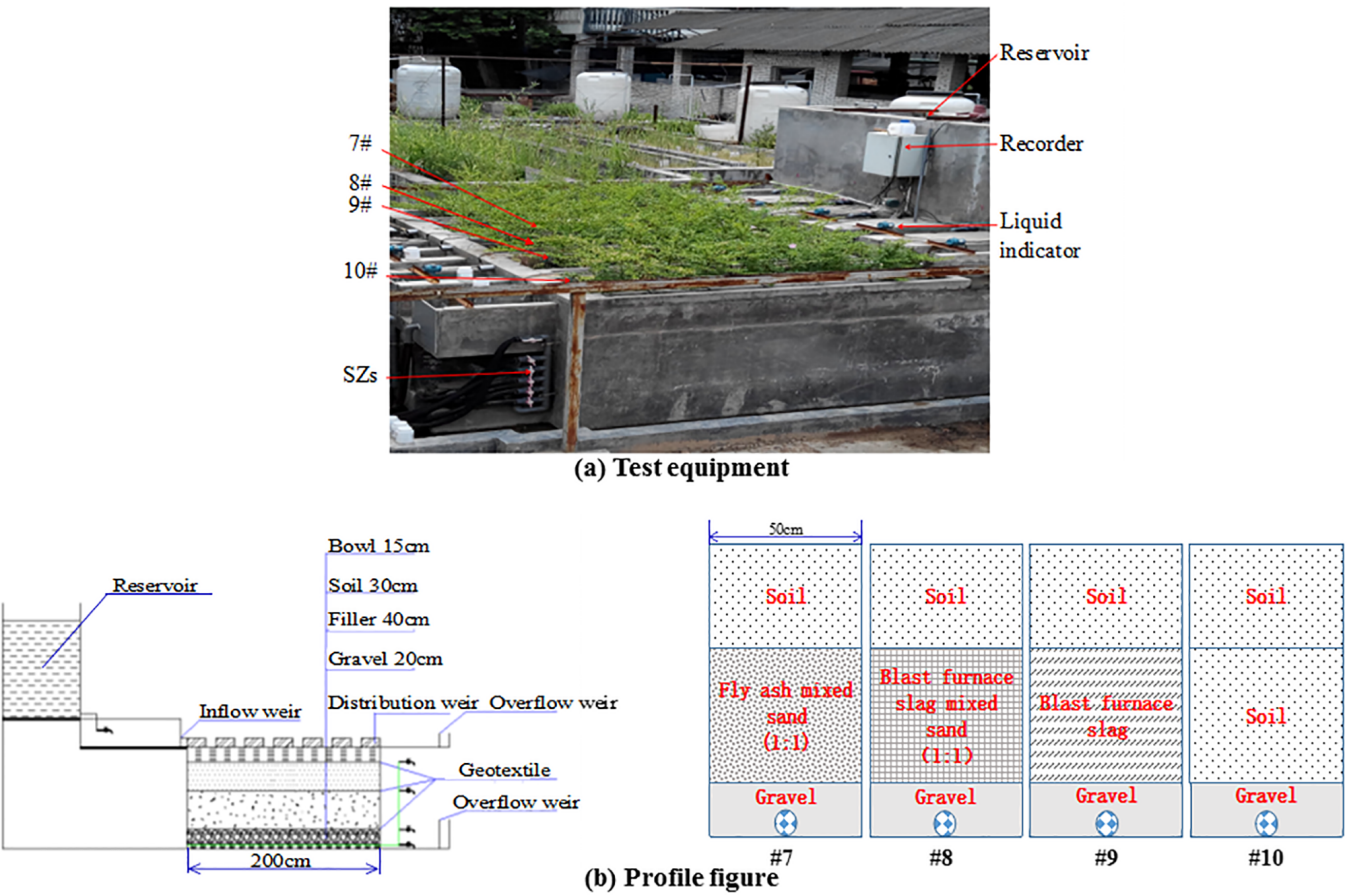
Test equipment and profile figure of bioretention tank. (a) Test equipment. (b) Profile figure.

Different group bioretention tanks were designed and constructed in the open-air testing ground of Xi’an University of Technology. Four bioretention tanks were constructed (Fig 1). The particle content (0.05~2 mm) of the soil of the four groups was above 90% (90.6%, 92.2%, 90.4%, 90.1%). Significant differences in runoff quality were recorded between non-vegetated and vegetated bioretention (*D*. *marginata*) facilities, with the latter producing better purification effect [29], and potential clogging could be prevented by vegetated root [30]. Therefore, the same plants used in local road greening (*Ligustrum quihoui Carr* and *Ophiopogogon japonicus*) were planted in the four tanks, since these two plants have a stronger drought and waterlogging resistance, they have been used as a road plant in Xi’an. The bottom of each bioretention tank featured an anti-seepage mechanism. In each bioretention tank, 30° triangle weirs were installed in the inflow, outflow and overflow ports. Water depth was observed with a water level recorder (Teng Hui temperature control instrument and meter plant in Yuyao, ZheJiang province), and flow was calculated according to the water depth before the weirs. The internal and external structures of the test tanks are shown in Table 1, in this study, device description was carried out in device number #7 –#10.

**Table 1:** Structure of bioretention tanks.

Device number	Filler	Plant	Mulch	Size
#7	Fly ash mixed with sand (volume ratio 1:1)	*Ligustrum quihoui Carr and Ophiopogogon japonicus*	*Platanus orientalis Linn* leaves	Length: 2.0 m Width: 0.5 m Depth: 1.05 m
#8	Blast furnace slag mixed with sand (volume ratio 1:1)			
#9	Blast furnace slag			
#10	Soil			

### Test program

#### Setting of water volume and quality

**Fig 2:**
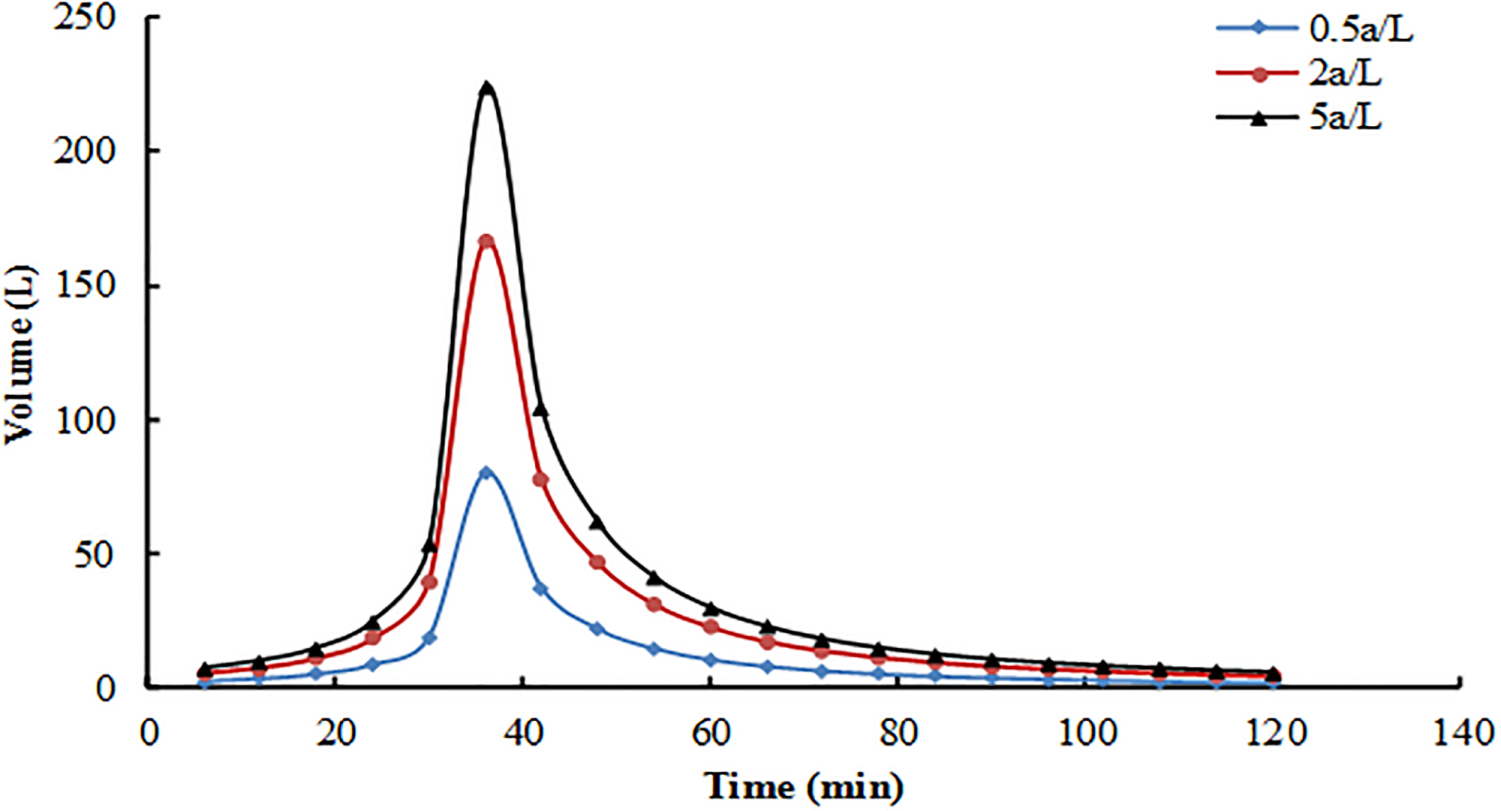
Rainfall pattern.

The ratio of the bioretention facilities surface area and the catchment area is 1:10–1:20 [31]. This ratio was set to 17:1 in the present study. Water volume was calculated with the Xi’an stormwater intensity formula shown in Eq (1) [32] and the design volume formula shown in Eq (2). Rainfall duration was 2 h (120 min). The design volumes corresponding to the three types of recurrence intervals were 0.5, 2 and 5 a. Short duration rainfalls showed a single-peak rain pattern, and most of the peak flow appeared in the previous period (the first half of a short period of rainfall). The effect was effectively observed when peak flow or storage volume was calculated with the Chicago pattern (The Chicago pattern is a common single peak rain type, it generally meets the requirement of precision, and it is easy to determine the process of heavy rain. It is widely used in both domestic and foreign countries, and it is recommended that the rain pattern is used as the design of rain pattern), with the factor of peak flow (the proportion of peak flow time of total time) at 0.3. Three recurrence intervals of the rain process line are shown in Fig 2. The calculation of water volume is shown in Table 2.

$$q=\frac{2785.833\times \left(1+1658\;{\text{log}}_{10}\;P\right)}{(t+16.813)0.9302}$$ (1)

$$Q_{s}=q\varphi F$$ (2)

where *q* is the rainfall intensity [L/(s·hm^2^)], *P* is the recurrence interval (a), *t* is the simulated rainfall duration (min), *Q*_*S*_ is the design flow (L/s), *φ* is the runoff coefficient, and *F* is the catchment area (hm^2^).

**Table 2:** Calculation of water volume.

*P* (a)	*t* (min)	*q* (L/s.ha)	*φ*	*F* (ha)	*Q*_*s*_ (L/s)	*V* (L)	Precipitation (mm)	Level
5	120	52.0922	0.9	0.0017	0.0797	573.8472	33.76	High
2	120	38.7762	0.9	0.0017	0.0593	427.1591	25.13	Middle
0.5	120	18.6300	0.9	0.0017	0.0285	205.2285	12.07	Low

Note: V is design volume.

The TP concentration of the actual rainwater quality of surface runoff in the middle and latter periods in Xi’an are 0.539–0.880 mg/L [33]. Synthetic stormwater was dosed with contaminants through a methodology similar to that used by Deletic and Fletcher [34] to replicate typical stormwater runoff pollutant found in urban runoff (S1 Table). This study increases the concentration configuration by two or three times. There are two pollution concentrations in this study, namely, high and low pollution loads, were tested in each of the four bioretention tanks (Table 3). In a single test, the influent pollution concentrations were constant (high or low pollution concentration). Although high pollution loads are not expected to occur naturally (aside from possibly resulting from extremely long antecedent dry periods, or pollutant loads can be high in heavy traffic areas or "hot spots"—parking lots, gas stations, etc.), they were included in this study to help identify any distinctive trend that may otherwise be difficult to measure [13].

**Table 3:** Concentration and synthetic additive of the testing water.

Pollutant	COD	NO_3_^-^-N	NH_3_-N	PO_4_^3-^	Cu	Zn	Cd
High concentration (mg/L)	600	14	6	2.5	1.0	1.5	0.05
Low concentration (mg/L)	300	8	3	1.0	0.5	0.8	0.03

#### Internal and external influencing factors

Three bioretention tanks (#7, #9 and #10) were selected for this part. Nine pilot tests were conducted to study the tank operation effect under the conditions of different internal [filler type and submerged zones (SZs), the SZs setting was designed to create anaerobic areas inside the device] and external (rainfall interval times and influent pollution concentrations) influencing factors. Among these nine tests, Test 1 served as the preliminary test. Tests 2 and 3 were performed to investigate the influence of SZs on P treatment, the depth of SZs in Test 2 is 150 mm. Tests 4, 5 and 6 were conducted to examine the influence of rainfall interval time on P treatment and soil restoration. Tests 7 and 8 were aimed at determining the influence of flow volume on P treatment, and Tests 8 and 9 were aimed at determining influent pollutant concentration in P treatment. Without considering the other parameters (TSS, N, metals, COD, pH, etc.). Except for Test 2, no SZs were set in the tests. The sets of early-stage test conditions were made to be as consistent as possible. The test information is shown in Table 4.

**Table 4:** Test for influencing factors.

Test number	Pollutant concentration	Submerged zone (mm)	Interval time (d)	Test date	Water volume
Test 1	Low	0		2015.5.20	5a (high)
			7		
Test 2	Low	150		2015.5.27	5a (high)
			7		
Test 3	Low	0		2015.6.30	5a (high)
			15		
Test 4	Low	0		2015.6.18	5a (high)
			7		
Test 5	Low	0		2015.6.25	5a (high)
			3		
Test 6	Low	0		2015.6.28	5a (high)
			7		
Test 7	Low	0		2015.7.05	2a (middle)
			7		
Test 8	Low	0		2015.7.12	5a (high)
			7		
Test 9	High	0		2015.7.19	5a (high)

#### Continuous operation test

During rainfall, bioretention facilities treat runoff pollution through adsorption and sedimentation of the filler layer. In the current work, the test was conducted to study the adsorption exhaustion point of three filler combinations. Without considering the other parameters, the adsorption means of P were studied under the condition of continuous inflow through the relationship between influent concentration and effluent concentration to determine the filler adsorption exhaustion point. Three tanks (#7, #9 and #10) were selected. Influent pollution was of low concentration. The influent volume schedule is shown in Table 5. Considering the continuous inflow for a long period, the filler was likely to lose its adsorption ability gradually after a few hours from low loads to high loads. Both influent pollutant concentrations and water volume were constant in each of the recurrence intervals [three recurrence intervals (0.5, 2, and 5 a) run 24, 12, 8 hours respectively, there are two hours of water distribution between recurrence intervals, and influent volume is constant throughout the test]. Influent samples were obtained every 2 h, and the effluent sample interval was 0.5 h. For a successive 3 h operation, the effluent concentration was equal to or greater than 90% of the average influent concentration, i.e. C_out_/[avg.C_in_×(1–10%)]≥1; in this case, the filler was considered to be exhausted. Influent and effluent accumulated pollutant loads were calculated, and pollutant accumulation in the system was determined. A background value analysis of the soil samples was also performed to evaluate three filler combinations of operating life of the system under continuous operation.

**Table 5:** Influent volume schedule for continuous operation.

Recurrence interval (a)	Precipitation (L/2h)	Running time (h)	Total influent volume (L)
0.5	205.23	24	2462.76
2	427.16	12	2562.96
5	573.85	8	2295.39

#### Intermittent operation test

During dry periods, the soil permeability of bioretention tanks recovers gradually. Through biological degradation, microbes break down organic matter, and plants absorb nutrients. In the present study, a water quality test was conducted under the condition of low influent concentration and high influent volume to explore changes and trends in the tank operation effect in the Xi’an climate, the precipitation in Xi’an is 573.7 mm, the rainfall distribution is mainly from June to September. A water volume test was also carried out under the condition of zero pollution and moderate influent volume. The interval time of each simulated rainfall test was 15 d.

#### Analysis method

Water quality analysis method: SRP was determined using a membrane filter and the molybdenum antimony anti-spectrophotometric method. TP was determined using potassium persulphate oxidation and the molybdenum antimony anti-spectrophotometric method [35].

Calculation analysis method: Concentration reduction efficiency (*CRE*) was calculated with Eq (3) for each simulated event as the percentage reduction in concentration with respect to the influent concentration for each pollutant (TP and SRP), no other water quality parameters (TSS, N, metals, COD, pH, etc.) were analyzed. It is important to note that this calculation for CRE is more valid when the flow rate, sampling interval, and concentration are constant. Otherwise event mean concentrations (*EMC*) were determined with Eq (4) for each test flow event, and efficiency ratios (*ER*) were calculated with Eq (5).

$$CRE=\frac{{\bar{C}}_{in}-{\bar{C}}_{out}}{{\bar{C}}_{in}}$$ (3)

$$EMC=\frac{M}{V}=\frac{\int_{0}^{t_{d}}Q_{\left(t\right)}C_{\left(t\right)}dt}{\int_{0}^{t_{d}}Q_{\left(t\right)}dt}\approx \frac{\sum_{\text{i}=1}^{\text{n}}C_{i}Q_{i}\Delta t_{i}}{\sum_{\text{i}=1}^{\text{n}}C_{i}Q_{i}\Delta t_{i}}$$ (4)

where *C*_*in/out*_ is the concentration of inflow or outflow (mg/L), $\bar{C}$ is the arithmetic mean concentration (mg/L), *Q*_*i*_ is the flow during period *i* (L/s), *C*_*i*_ is the concentration associated with period *i* (mg/L), and *n* is the total number of aliquots collected during the event.

$$ER=\frac{EMC_{in}-EMC_{out}}{EMC_{in}}$$ (5)

In this test, the filler media shifted gradually from unsaturated to saturated, and the reservoir was always maintained. Filler depletion was defined as follows: tank operates continuously for 3 h and effluent target pollutant concentration (SRP) is equal to or greater than the average SRPin ×(1–10%). Tanks #7, #9 and #10 were operated continuously for 48 h, and the influent concentration was constant and then gradually changed from small hydraulic load to large hydraulic load. The volume of water hydraulic loading in the reservoir was set as follows: small running flow for 24 h and running times of 12 and 8 h for middle and high volumes respectively.

**Fig 3:**
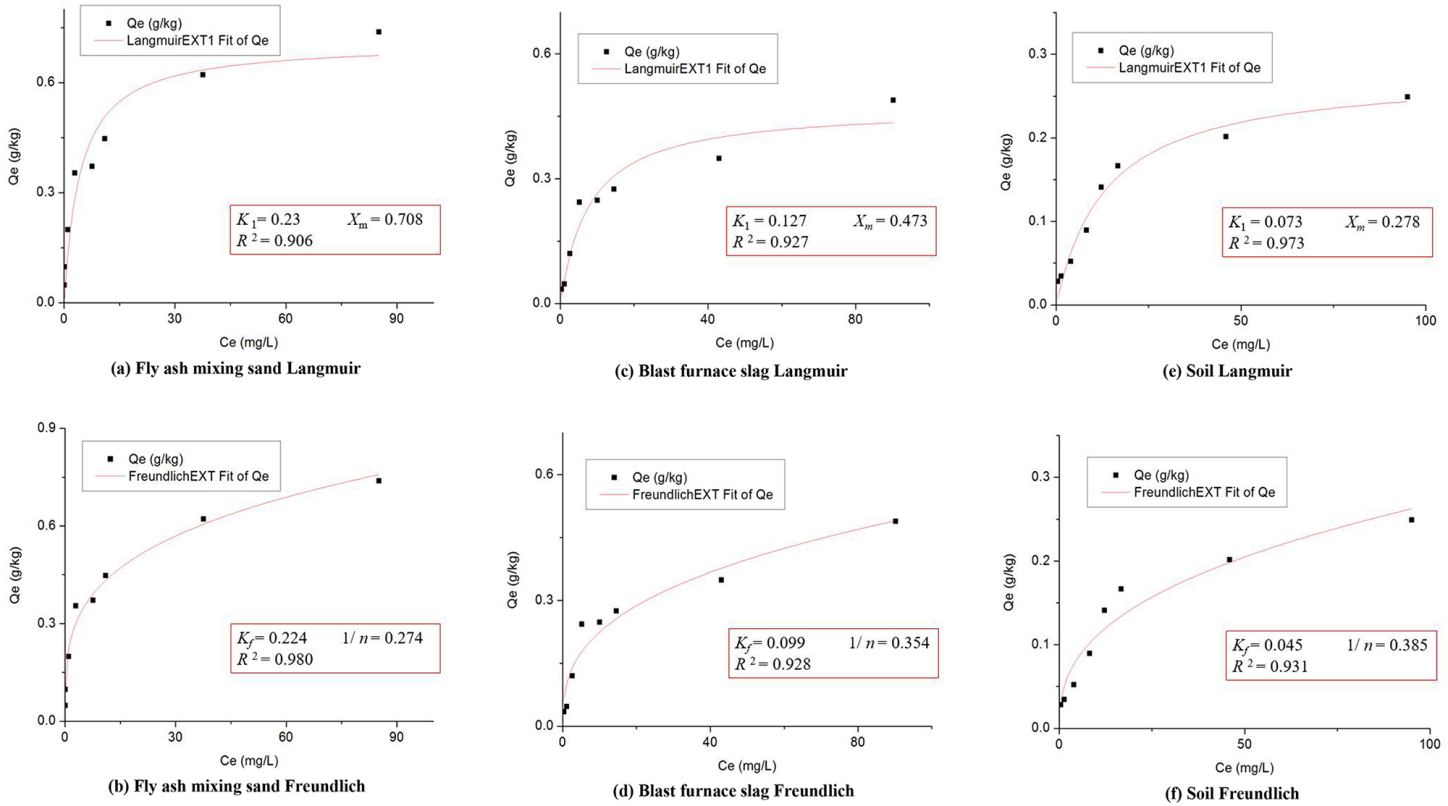
Adsorption isotherm. (a) Fly ash mixing sand Langmuir. (b) Fly ash mixing sand Freundlich. (c) Blast furnace slag Langmuir. (d) Blast furnace slag Freundlich. (e) Soil Langmuir. (f) Soil Freundlich.

In the statistical analysis of SRP removal performances of the bioretention tanks, only one dependent variable *y* (*ER*_SRP_) was used. The independent variables (*x*) included the concentration of influent pollutants, water hydraulic load, rainfall interval time, SZs, and filler combinations type. Langmuir and Freundlich sorption isotherm of three filler combinations (#7, #9 and #10) are shown in Fig 3. The conclusion of two sets of equations are consistent. Therefore, the two parameters of Langmuir sorption isotherm equation (*X*_*m*_ and *K*_*1*_) are used to represent filler combinations type. Eq (6) and the primitive variable Eq (7) in the PLS model were standardized as follows:

$$y=a_{1}x_{1}+a_{2}x_{2}+a_{3}x_{3}+a_{4}x_{4}+a_{5}x_{5}+a_{6}x_{6}$$ (6)

$$y^{*}=b_{0}+b_{1}x_{1}+b_{2}x_{2}+b_{3}x_{3}+b_{4}x_{4}+b_{5}x_{5}+b_{6}x_{6}$$ (7)

where *y* is *ER*_SRP_ (%), *x*_*1*_ is the concentration of influent pollutants, mg/L; *x*_*2*_ is the water hydraulic load, L; *x*_*3*_ is the rainfall interval time, day; *x*_*4*_ is the height of SZs, mm; *x*_*5*_ is *K*_*1*_, L/kg, it can reflect the adsorption bond strength; and *x*_*6*_ is *X*_*m*_, mg/g, it is saturation adsorption capacity.

## Results and analysis

### Operation effect at different internal and external influencing factors

**Fig 4:**
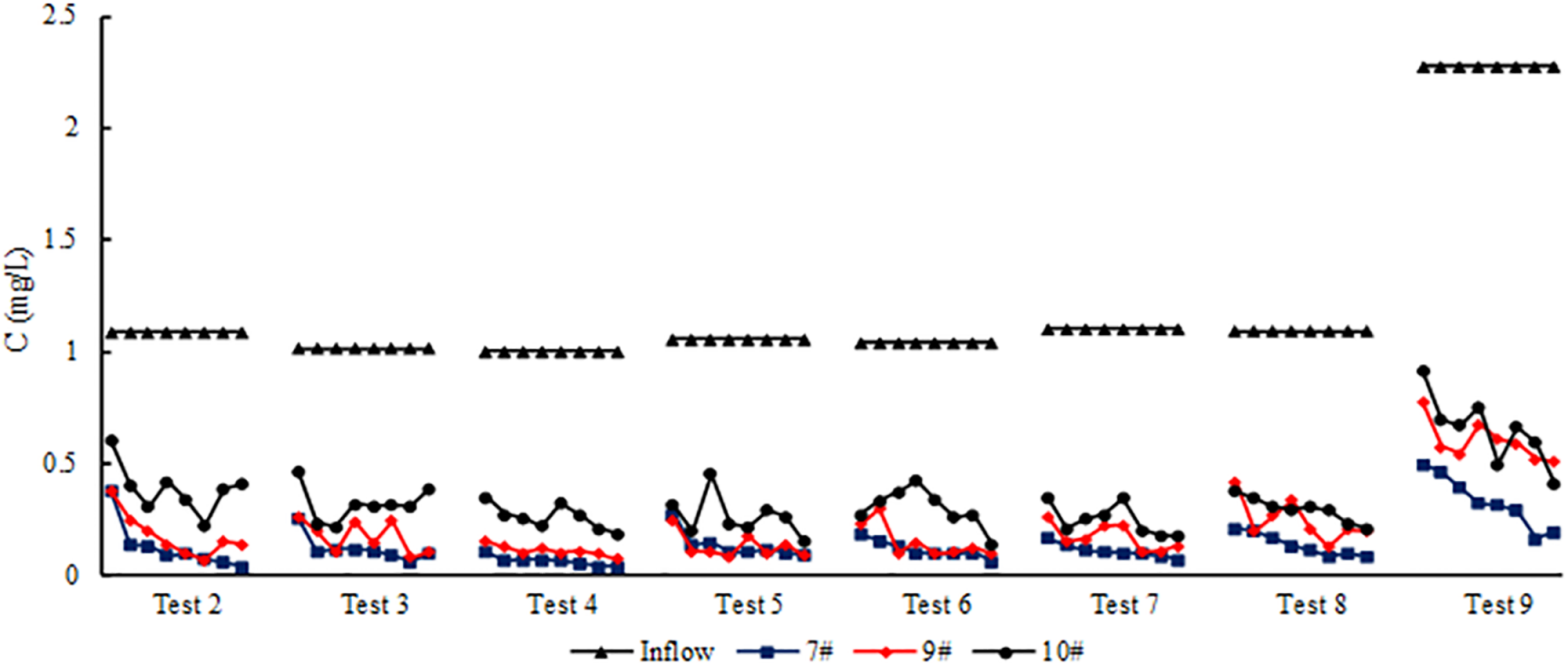
Concentration process lines of SRP.

The SRP pollutant concentration and reduction efficiency of three filler combinations of bioretention facilities were studied under the condition of whether to set SZs, different rainfall interval times, different influent hydraulic loads and two influent concentrations (high and low). The influent volume of Test 7 was moderate (P = 2a), whereas that of the others was high (P = 5a). The influent pollutant concentration of Test 9 was high, whereas that of the others was low. Therefore, the influent pollutant concentration of Test 7 was small, and that of Test 9 was large. The results of Tests 2 to 9 are listed in Table 6, the concentration process lines of influent and effluent SRP pollutants is shown in Fig 4.

**Table 6:** Test results of internal and external influencing factors.

Test number	Inflow/Outflow	SRP *EMC* (mg/L)	*ER*_SRP_ (%)
Test 2	Inflow	1.09±0.1	
	#7 outflow	0.123	88.7
	#9 outflow	0.218	80.0
	#10 outflow	0.4	63.6
Test 3	Inflow	1.014±0.079	
	#7 outflow	0.118	88.3
	#9 outflow	0.181	82.1
	#10 outflow	0.322	68.3
Test 4	Inflow	1.005±0.12	
	#7 outflow	0.066	93.5
	#9 outflow	0.119	88.2
	#10 outflow	0.268	73.4
Test 5	Inflow	1.057±0.08	
	#7 outflow	0.136	87.2
	#9 outflow	0.177	83.2
	#10 outflow	0.313	70.4
Test 6	Inflow	1.042±0.132	
	#7 outflow	0.121	88.3
	#9 outflow	0.18	82.7
	#10 outflow	0.317	69.5
Test 7	Inflow	1.105±0.112	
	#7 outflow	0.118	89.3
	#9 outflow	0.175	84.1
	#10 outflow	0.258	76.6
Test 8	Inflow	1.092±0.078	
	#7 outflow	0.134	87.7
	#9 outflow	0.259	76.3
	#10 outflow	0.304	72.2
Test 9	Inflow	2.279±0.154	
	#7 outflow	0.338	85.2
	#9 outflow	0.661	80.0
	#10 outflow	0.703	69.1

Note: ±value is standard deviation.

As shown in Table 6, the SRP pollution load reduction efficiency ranged from 85.2% to 93.5% for test #7; from 80.0% to 88.2% for #9; and from 63.6% to 76.6% for #10, respectively. Among the three studied filler combinations, the media with fly ash mixed with sand achieved the best P pollution reduction efficiency. The soil showed poor P removal. Compared with the change in P removal of the tanks in Test 2, the change in Test 3 was not large. Moreover, the setting of SZ exerted minimal influence on the P removal performances of the three filler combinations. However, Palmer et al. [25] found that SRP reduction was significantly better in columns without SZs (80%) than in columns with SZs (67%), the depth of SZ was 30.5 cm (12 in), it covered small gravel mineral aggregate mix. In the present study, the depth of SZ was 150 mm, and the gravel particle size was 1.5–3.0 cm. The difference of SZs depth is about 150 mm, and compared with small particle size mix (the former), the adsorption performance of the latter might be poor. A comparison of Test 4 with Tests 5 and 6 showed that the P removal exhibited a slight decline. The extension of the dry period slightly promoted the operation effect; this result is almost the same as that obtained by Hatt et al. [36] and Blecken et al. [37], who did not observe significant relationships between the treatment of P, sediments, heavy metals and the dry period. However, the outflow concentrations of N were significantly higher upon re-wetting following extended dry periods in comparison with re-wetting following wet periods, this also means that N is different from P removal mechanism, the change of oxygen environment in the system is very important for the removal of nitrogen. Test 7 involved minimal pollution load, and Test 9 involved a large pollution load. Compared with that in Test 7, the mean concentration reduction efficiency of tank #7 for the SRP event was still higher than 85% in Test 9. This result showed that at a certain concentration range, the removal ability of fly ash mixed with sand was extremely strong despite the improvement of pollution load of the bioretention facilities. This removal ability satisfies the requirement of urban surface runoff treatment. This combination can be used to purify surface runoff with high P pollution concentration. Fly ash has a porous structure and large specific surface area, so it has strong adsorption capacity, it is also used in other types of SCMs. Hwang et al. [38] used fly ash to optimise pervious concrete pavements and integrated them into bioretention facilities; they then determined the stormwater runoff volume reduction and water quality performance. Consequently, in the process of bioretention filler selection under the condition of high hydraulic and pollutant loads, screening out filler types with high adsorption performance or improving filler P adsorption capacity is crucial.

### Analysis of continuous operation test

A breakthrough curve analysis was conducted to study the filler exhaustion point. The accumulation of inflow and pollutant loads was examined to assess the operating capacities of the tanks. The volumetric weight and water content of soil and filler materials, soil texture and chemical properties of pollutants affect the migration characteristics of runoff pollutants in soil.

**Fig 5:**
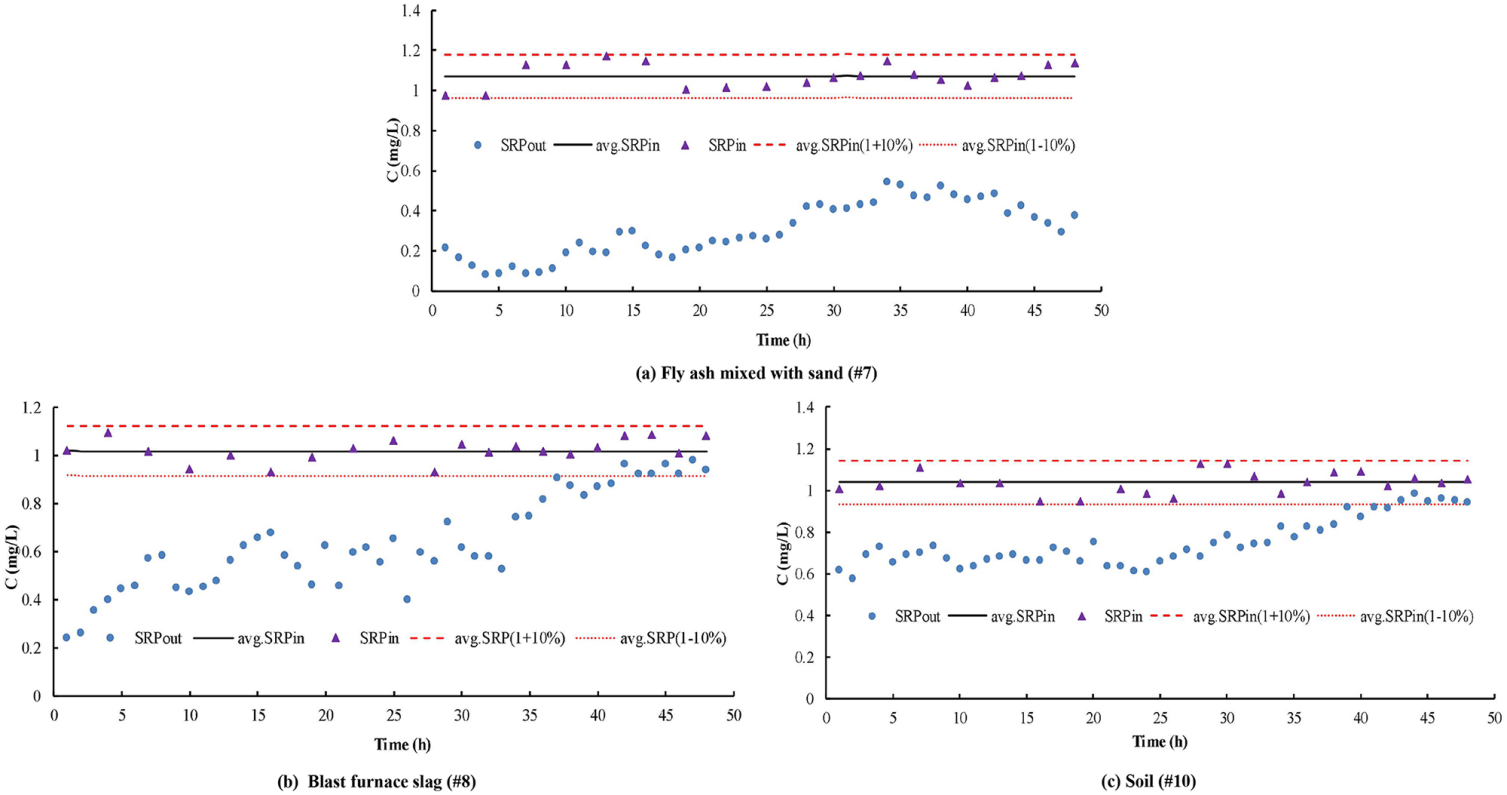
Exhaustion test result. (a) Fly ash mixed with sand (#7). (b) Blast furnace slag (#8). (c) Soil (#10).

As shown in Fig 5(a), in the case of continuous draining for 48 h, the tank containing fly ash mixed with sand consistently failed to reach the exhaustion point, and the SRP was of low concentration. The water quality test with a short duration (2 h) revealed that the purification effect of this filler combination on SRP remained strong under short- or long-term simulated rainfall duration. As shown in Fig 5(b), in the blast furnace slag tank operating for 42 h, the effluent concentration C exceeded the average SRP_in_ ×(1–10%) for 3 h. In the water quality test with a short rainfall duration, the blast furnace slag presented a strong purification ability, but its continuous purification effect on SRP was poor in the long rainfall duration condition. As shown in Fig 5(c), in the soil tank operating for 41 h, the effluent concentration C exceeded the average SRP_in_ ×(1–10%) for 3 h. The early stage of SRP was in the condition of high effluent concentration. Unlike those of tank #7, the short- and long-term purification abilities of the soil tank were weak.

When the concentration of SRP reached the exhaustion point, the rainfall volumes of tanks #9 and #10 corresponding to the 17 m^2^ catchment area were 331.2 and 314.26 mm respectively; compared with the average annual rainfall of Xi’an (573.7 mm), precipitation from the beginning to the exhaustion point in the process was equivalent to 0.58 and 0.55 a (annual). O’Neill and Davis [39] studied P adsorption capacity using a mini column with an inner diameter of 2.5 cm. The dissolved P concentration of the influent was 120 μg/L, and two groups of bioretention systems [the filler was soil without special media: water treatment residuals (WTRs), hardwood bark mulch, or leaves compost] were obviously exhausted. The two groups reached the exhaustion point, and the water volumes were 220 and 250 mm precipitation of the 20:1 catchment ratio. These values were equivalent to South Carolina state’s rainfall of 0.23 a. However, the amended filler (added with special media) reached the exhaustion point, and the accumulative rainfall was tens of times more than the first two sets of devices. Soleimanifar et al. [20] demonstrated that WTR-coated mulches are new, low-cost and effective filter media for urban stormwater treatment, thereby providing a sustainable approach to reuse industrial waste for environmental pollution control. Therefore, adding a certain proportion of special media into fillers is also an effective measure of P removal.

### Analysis of intermittent operation test

**Fig 6:**
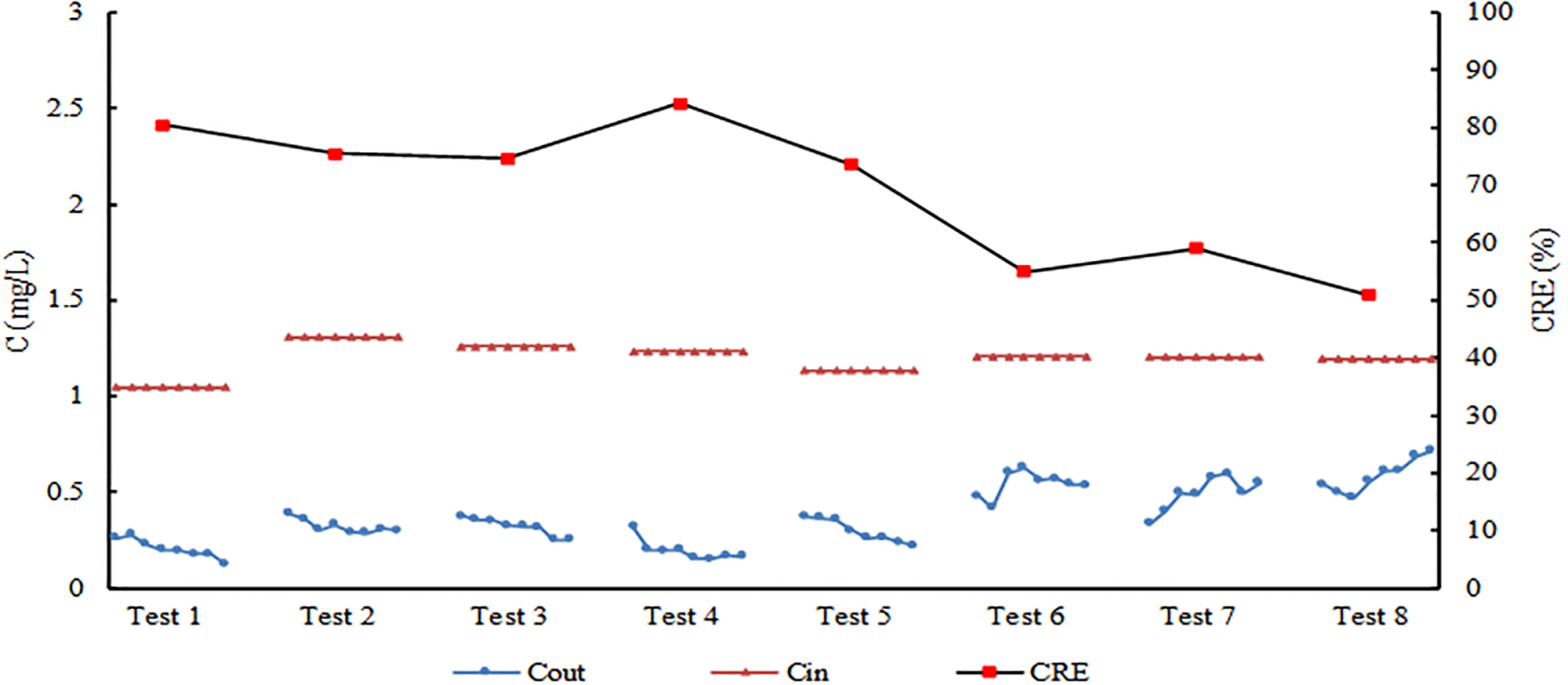
Reduction efficiency and concentration process lines of SRP (#9).

The results of eight tests and the concentration process lines of SRP pollutants for #8 are shown in Fig 6. The water quality purification effect in eight months revealed that the CRE of SRP ranged from 50.9% to 84.2%. The three CREs exhibited significant fluctuation. Over time, the purification effects showed a decline. The purification ability of #8 was considerably strong from June to September. With the change of seasonal temperature from October to December, the P pollutant purification ability of the tank decreased, with the reduction efficiencies below 60%. In the eight events (from May to December) of the intermittent operation tests, the change in the removal of SRP concentration in the effluent was evident, and the effluent concentrations of the SRP presented different regulations. In the study of Blecken et al. [37], P (mainly for particles) was not influenced by the low-temperature laboratory experiment (column, 3.77 cm diameter). Differences in study results are possibly due to the P species and test scale, such as TSS removal is not significantly affected by temperature, it is removed by filtration process which depend on permeability and type of fills media [40].

Although the P removal effects via bioretention across four seasons are stable, low temperatures may affect plants and microorganisms in bioretention facilities. Such conditions may weaken the removal efficiency of bioretention for other pollutants. Therefore, the design of a mulch layer is important. An improved mulch design can effectively isolate bioretention facilities from the external environment and lead to surface conditions that benefit plant seedlings and seed germination. A good mulch material must exhibit the following characteristics: 1) fully decomposed so as not to produce secondary organic loading in facilities, 2) a circumneutral pH, 3) fluffy structure with high fiber content to provide good thermal insulation, 4) good contact between the seed and the mulch for germination and 5) good moisture holding capacity [41].

In this study, the bioretention systems relied on vegetation and soil, filler and zero compost to treat stormwater runoff. No P leaching occurred in the eight intermittent operation tests. Mullane et al. [27] demonstrated that intermittent rainstorms release N, P and Cu in leachate from compost in bioretention systems, compost can serve as a sustainable source of leaching of nutrients and metals. However, Takaijudin et al. demonstrated that *Elaeis Guineensis* leaves compost is recommended to be used as part of engineered soil media due to its capabilities in removing Ammoniacal Nitrogen (NH_3_-N) [42]; hence, the selection of carbon source types for bioretention design is particularly important. Hsieh and Davis [43,44] stressed that care should be taken when using compost in areas with nutrient discharge, because although compost can be beneficial for removing certain pollutants, such as metals [45,46], its decomposition may result in the net leaching of P from the media [9].

### Partial least squares regression (PLS) modeling analysis

Eighteen groups of data from seven rainfall events were considered as modeling samples. Tests 1, 3 and 8 were used in the model examination, and the modeling and examination samples are listed in Tables 7 and 8. The standardized variable Eq (8) is as follows:

$$y=-0.1382x_{1}-0.0473x_{2}+0.1098x_{3}-0.0765x_{4}+0.442x_{5}+0.4557x_{6}$$ (8)

**Table 7:** Model fitting parameters and measured values.

Test number	Device number	SRP *C*_*in*_ (mg/L)	*V*_*in*_ (L)	Rainfall interval time (d)	SZs (mm)	*K*_*1*_ (L/kg)	*X*_*m*_ (g/kg)	*ER*_SRP_ (measured) (%)
Test 2	#7	1.09	536.49	7	150	0.23	0.708	88.7
	#8	1.09	535.11	7	150	0.127	0.473	80.0
	#10	1.09	532.53	7	150	0.073	0.278	63.6
Test 4	#7	1.095	525.88	15	0	0.23	0.708	93.5
	#8	1.095	539.16	15	0	0.127	0.473	88.2
	#10	1.095	525.43	15	0	0.073	0.278	73.4
Test 5	#7	1.057	532.82	7	0	0.23	0.708	87.2
	#8	1.057	525.21	7	0	0.127	0.473	83.2
	#10	1.057	515.11	7	0	0.073	0.278	70.4
Test 6	#7	1.042	546.71	3	0	0.23	0.708	88.3
	#8	1.042	533.03	3	0	0.127	0.473	82.7
	#10	1.042	524.76	3	0	0.073	0.278	69.5
Test 7	#7	1.105	353.76	7	0	0.23	0.708	89.3
	#8	1.105	361.49	7	0	0.127	0.473	84.1
	#10	1.105	359.63	7	0	0.073	0.278	76.6
Test 9	#7	2.279	518.86	7	0	0.23	0.708	85.2
	#8	2.279	517.71	7	0	0.127	0.473	80.0
	#10	2.279	503.54	7	0	0.073	0.278	69.1

**Table 8:** Test samples of measured and predicted SRP concentration reduction efficiencies and influencing factors.

Test number	Device number	SRP C_in_ (mg/L)	V_in_ (L)	Rainfall interval time (d)	SZs (mm)	*K*_*1*_ (L/kg)	*X*_*m*_ (g/kg)	*ER*_SRP_ (measured) (%)	*ER*_SRP_ (predicted) (%)
Test 1	#7	1.098	529.73	7	0	0.23	0.708	88.7	90.6
	#8	1.098	532.14	7	0	0.127	0.473	77.3	79.3
	#10	1.098	520.69	7	0	0.073	0.278	65.3	72.0
Test 3	#7	1.014	526.03	7	0	0.23	0.708	88.3	90.8
	#8	1.014	531.52	7	0	0.127	0.473	82.1	79.6
	#10	1.014	520.81	7	0	0.073	0.278	68.3	72.2
Test 8	#7	1.092	507.05	7	0	0.23	0.708	87.7	90.7
	#8	1.092	525.44	7	0	0.127	0.473	76.3	79.4
	#10	1.092	534.41	7	0	0.073	0.278	72.2	71.9

The primitive variable Eq (9) is as follows:

$$y^{*}=65.9478-2.6405x_{1}-0.0064x_{2}+0.2618x_{3}-0.0117x_{4}+58.0812x_{5}+22.1841x_{6}$$ (9)

**Fig 7:**
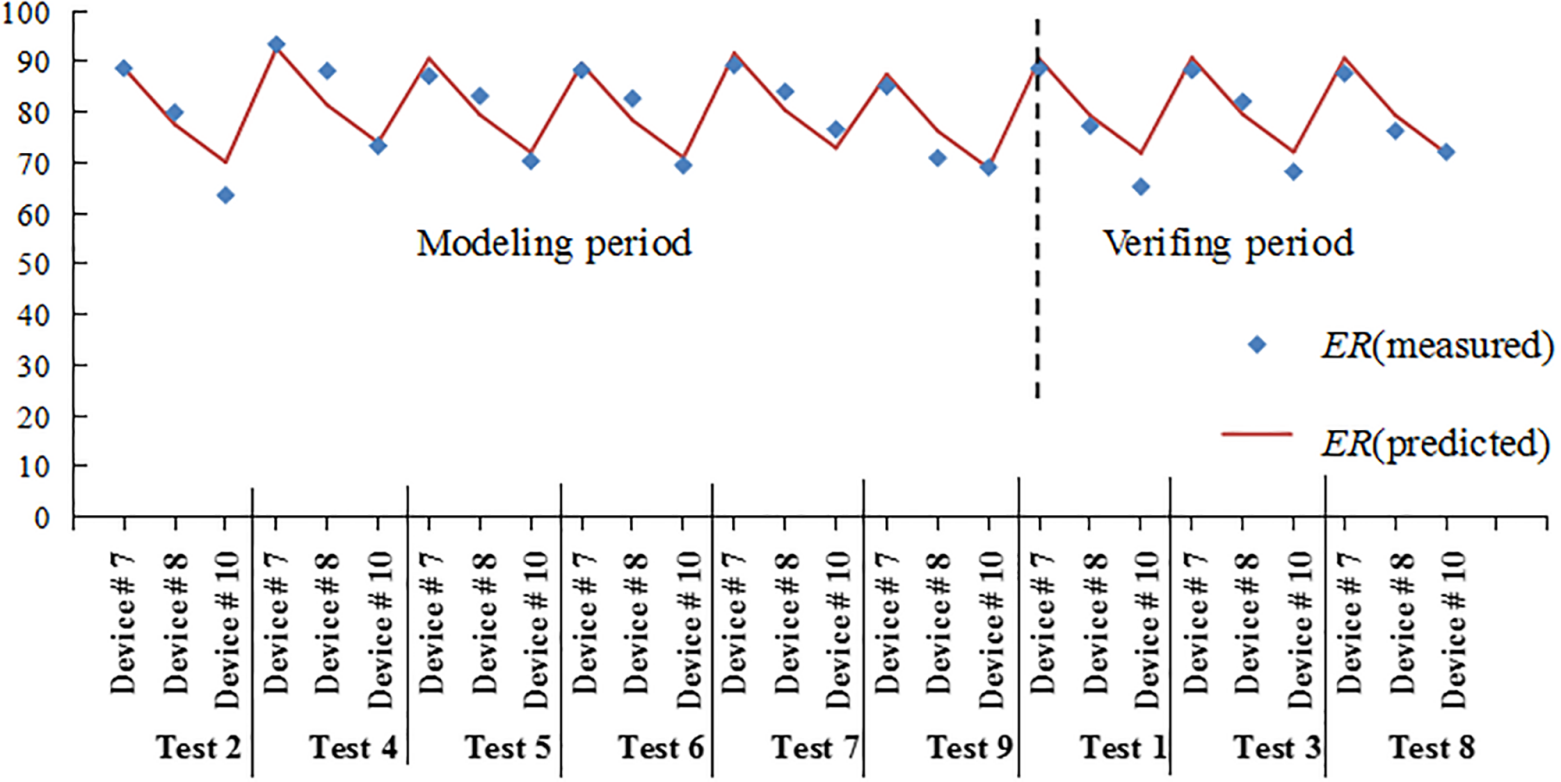
Comparison of measured and predicted values.

**Fig 8:**
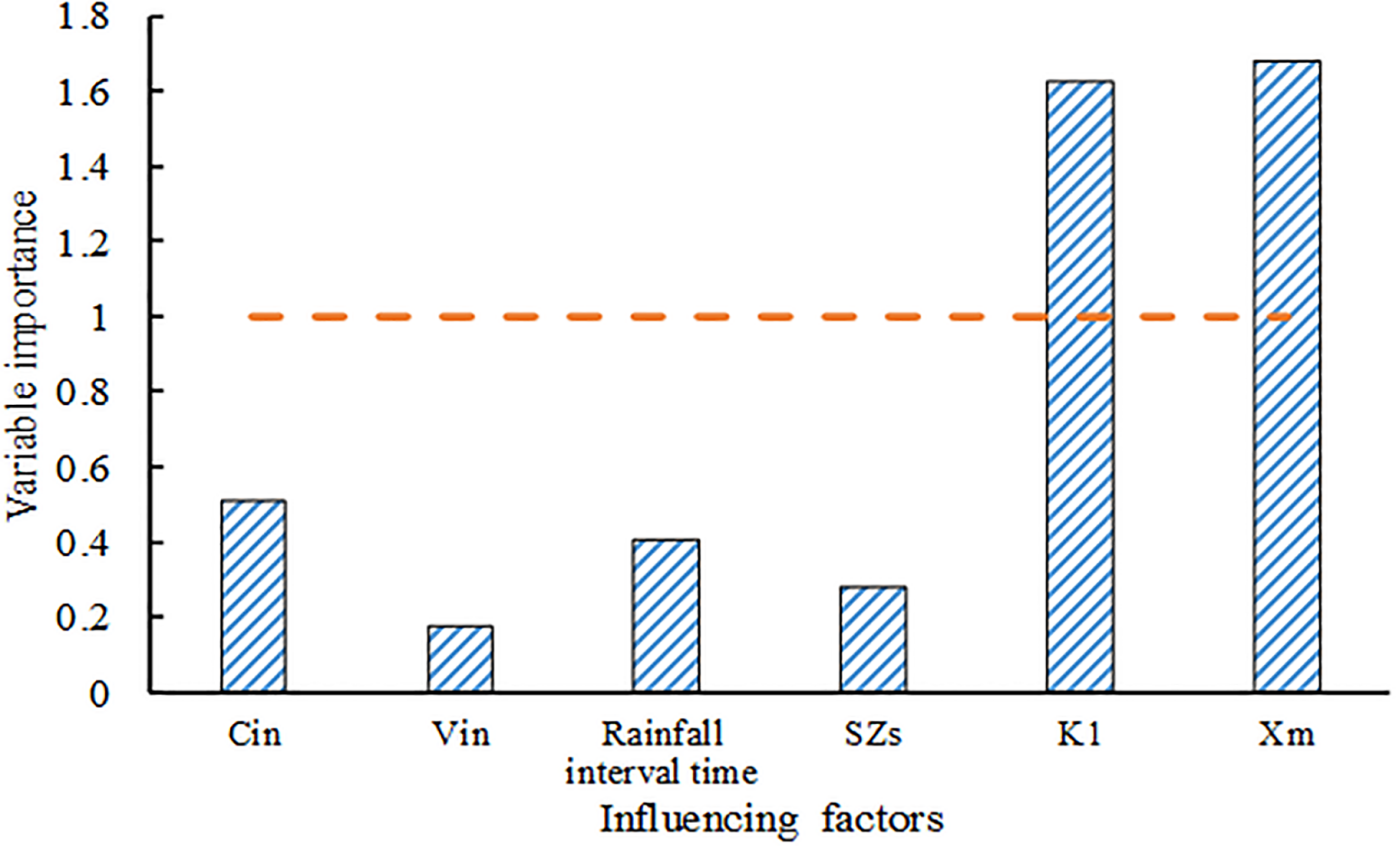
Analysis of the importance of influencing factors.

**Fig 9:**
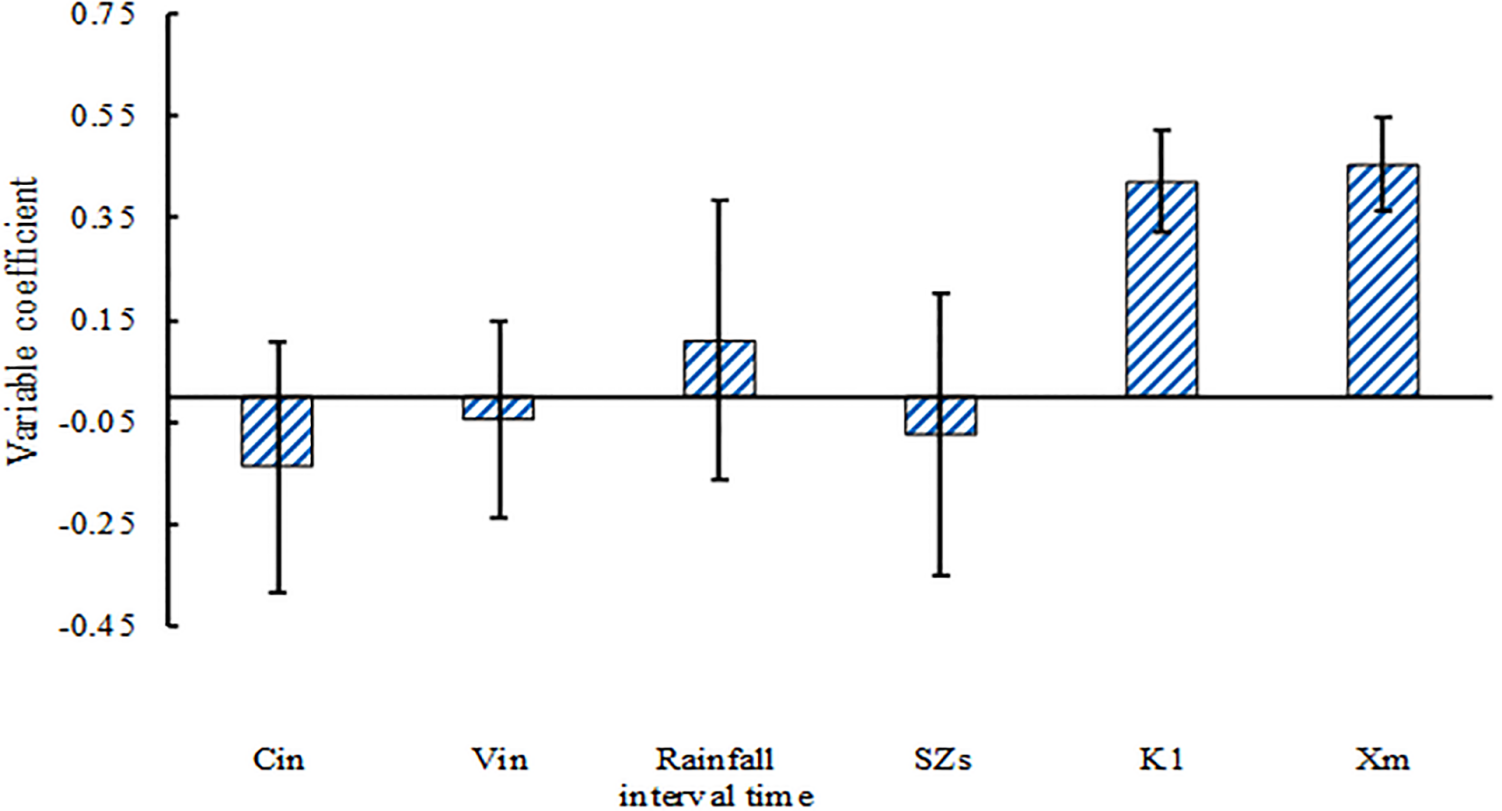
Correlation and standard deviation analysis of removal rate and influencing factors.

The difference between predicted and measured values of SRP removal was analyzed using PLS (Fig 7). The regression line for the observed values showed better fitting with deterministic coefficient R2 of 0.849 and Nash-Sutcliffe efficiency coefficient of 0.841. As shown in Fig 8, the result from the PLS analysis between SRP removal rate and influencing factors showed that adsorption bond strength (VIP = 1.628) and saturation adsorption capacity (VIP = 1.678) were the most important influencing factor for SRP removal in the bioretention facilities. Some reports showed that with increasing filler depth, bioretention facilities perform effectively in terms of TP removal [45,47]. The order of importance in other factors is as follows: inflow concentration, rainfall interval time, the height of SZs, and inflow water volume. Five important factors were selected in the study. Rainfall interval time, inflow concentration, and inflow water volume were considered as the external influencing factors for bioretention tanks. The filler type and SZs were considered as the internal influencing factors. Correlation and standard deviation analysis (Fig 9) showed a positive relationship between SRP removal and adsorption bond strength, saturation adsorption capacity, and rainfall interval time. However, negative relationship was found between SRP removal and the height of SZs, inflow concentration, and inflow hydraulic loading. It is noteworthy that there are fewer modeling samples, the study also needs to consider other related research conclusions.

## Conclusions

Through three series of different tests involving influencing factors, continuous operation and intermittent operation, this study revealed that the combination of fly ash mixed with sand achieved the best pollution reduction efficiency. The setting of the SZ exerted minimal influence on the P removal of the three filler combinations. The extension of the dry period slightly promoted the P purification effect. The combination of fly ash mixed with sand demonstrated a positive purification effect on the SRP during short- or long-term simulated rainfall duration. Blast furnace slag also presented a positive purification effect in the short term, although its continuous purification effect on the SRP was poor in the long term. The short- and long-term purification abilities of soil were both weak. Under intermittent operations across different seasons, the SRP removal was unstable, and the effluent concentration processes were different. The mean concentration removal of the SRP event was positively related to the adsorption capacity of filler and rainfall interval time, and the adsorption capacity of filler (adsorption bond strength and saturation adsorption capacity) was the most important factor that affected the SRP removal. By contrast, a negative relationship was observed between SRP concentration removal and submerged zones, influent concentration and volume.

## Supporting information

S1 Table The synthetic additive for configuring contaminants. (DOCX)
