# Supplementary material for: Experimental study and simulation of phosphorus purification effects of bioretention systems on urban surface runoff
Source: PLoS One. 2018 May 9;13(5):e0196339. doi: 10.1371/journal.pone.0196339 (PMC5942788; doi:10.1371/journal.pone.0196339)
Supplement: S1 Table — (DOCX) [file pone.0196339.s001.docx]

## Appendix

S1 Table. The synthetic additive for configuring contaminants

| Pollutant | COD | NO_3_^-^-N | NH_3_-N | PO_4_^3-^ | Cu | Zn | Cd |
| --- | --- | --- | --- | --- | --- | --- | --- |
| Synthetic additive | C_6_H_12_O_6_ | KNO_3_ | NH_4_Cl | KH_2_PO_4_ | CuCl_2_ | ZnSO_4_ | CdCl_2_ |
